# Worthless Drugs

**Published:** 1875-11

**Authors:** E. H. Gibbs


### WORTHLESS DRUGS.

Twenty years ago the principal hospital of a large city was in charge of two eminent physicians now resident in New York. I was at that time one of the "Internes," or house physicians, of the hospital, and accompanied the older doctors in their daily visits through the wards. Their prescriptions were almost always very simple: the variety of medicines prescribed in the various cases under treatment probably not exceeding a dozen.

During every summer these physicians would go to the country for a few weeks; and while absent their places were supplied by other physicians, who seemed to almost exhaust the pharmacopœia in search for drugs to fill their prescriptions. It would, indeed, have required an impossible increase of our stock of medicines to make up their prescriptions; and we did the only thing we could do under the circumstances—disregarded altogether the directions, and gave what we knew the regular physicians would have prescribed,—so that there was little opportunity to compare the merits of the different methods; but it is certain that there was no hospital in the country were diseases were more successfully treated, or the rates of deaths less.

I recently called at a drug-store, and asked to see a pharmacopœia. Instead of the well-remembered volume, nearly as large as a family Bible, which I had expected to see, a small octavo volume of about two hundred pages was handed me; and that contained all and more than all that was worth preserving, of the thousands of so-called "remedies." The compiler of that book has taken a step in advance of the age; but the time is coming when even this small edition will be greatly abridged. The popular idea that for every ailment nature has provided a remedy is absurd: most roots and herbs that are classed as medicines produce no appreciable effect on the system either in health or sickness; and of those that do, one or two of the best of each variety could be selected, and the rest cast aside as worthless. Their very multitude produces confusion in the minds of medical men, who often select those illy adapted to the case under treatment. Were a competent commission of chemists and physicians to select a few of the best, with authority to prohibit the use of all others, humanity would be gainers. The multitude of remedies recognized by the medical profession serve as a protection to charlatanism. There is need of but few, and the nature and properties of these should be understood by every intelligent person. When the physician is called he should not be permitted to shield his ignorance behind an assumed dignity, or disguise it with a flow of incomprehensible medical terms, or conceal it in prescriptions written in bad Latin. He should be subjected to a rigid examination, to ascertain if he understands the disease he is called on to treat; and if so, what he proposes to do, and why. The general adoption of such a course by intelligent people would weed out the quacks who have degraded the profession, and who are responsible for much misery.

A person must be educated to any sort of business to make it successful, except that of medicine. In this, modest merit has a poor chance in competition with ignorance and brazen assurance. That these things are so is the fault of people who suffer, and endure them.

It is an anomaly, that, in matters of ordinary business, where property is involved, business competency is always demanded; but we place our lives and the lives of our dear ones at the mercy of some ignorant pretender, because we have a vague impression that he is a successful doctor. As before remarked, the only cure for this evil—for it is an evil of fearful magnitude, entailing lifelong suffering on thousands—is to hold physicians to a strict responsibility. Compel them to throw off all mystery in the presence of patients and their friends. Any physician who insists on assuming the "air mysterious," who can not give a reason for his recommendations, and who will not, when desired, write out in language which patients and attendants can clearly understand, his prescriptions, is more or less a sham, and is therefore unworthy of confidence. It ought to be the law that the physician should write his diagnosis on his prescription, instead of leaving his conclusion as to the disease, to be guessed at from the drugs employed. We do not care how speedily the drug-list is reduced, nor how effectively. A consistent life is of more value than all the medicines under the sun.

E. H. Gibbs, M.D.

---

Marriage is the normal and legitimate order of society, and whatever tends to prevent marriage tends to destroy that order, to disrupt the true social bond, and to open the door for immorality.

